# Cyclin B3 Deficiency Impairs Germline Stem Cell Maintenance and Its Overexpression Delays Cystoblast Differentiation in *Drosophila* Ovary

**DOI:** 10.3390/ijms19010298

**Published:** 2018-01-19

**Authors:** Dongsheng Chen, Lijuan Zhou, Fuling Sun, Mingzhong Sun, Xiaoqian Tao

**Affiliations:** 1 Provincial Key Laboratory of the Conservation and Exploitation Research of Biological Resources in Anhui, College of Life Sciences, Anhui Normal University, Wuhu 241000, China; zhoulijuan@ahnu.edu.cn (L.Z.); sunfuling@ahnu.edu.cn (F.S.); sunmingzhong@ahnu.edu.cn (M.S.); taoxiaoqian@ahnu.edu.cn (X.T.); 2 The Institute of Bioinformatics, College of Life Sciences, Anhui Normal University, Wuhu 241000, China

**Keywords:** *cyclinB3*, germline stem cell, maintenance, overexpression, cystoblast, differentiation, *Drosophila*, ovary

## Abstract

It is well known that *cyclin* *B3* (*cycB3*) plays a key role in the control of cell cycle progression. However, whether *cycB3* is involved in stem cell fate determination remains unknown. The *Drosophila* ovary provides an exclusive model for studying the intrinsic and extrinsic factors that modulate the fate of germline stem cells (GSCs). Here, using this model, we show that *Drosophila* *cycB3* plays a new role in controlling the fate of germline stem cells (GSC). Results from *cycB3* genetic analyses demonstrate that *cycB3* is intrinsically required for GSC maintenance. Results from green fluorescent protein (GFP)-transgene reporter assays show that *cycB3* is not involved in *Dad*-mediated regulation of Bmp signaling, or required for *dpp*-induced *bam* transcriptional silencing. Double mutants of *bam* and *cycB3* phenocopied *bam* single mutants, suggesting that *cycB3* functions in a *bam*-dependent manner in GSCs. Deficiency of *cycB3* fails to cause apoptosis in GSCs or influence cystoblast (CB) differentiation into oocytes. Furthermore, overexpression of *cycB3* dramatically increases the CB number in *Drosophila* ovaries, suggesting that an excess of *cycB3* function delays CB differentiation. Given that the *cycB3* gene is evolutionarily conserved, from insects to humans, *cycB3* may also be involved in controlling the fate of GSCs in humans.

## 1. Introduction

**Figure 1:**
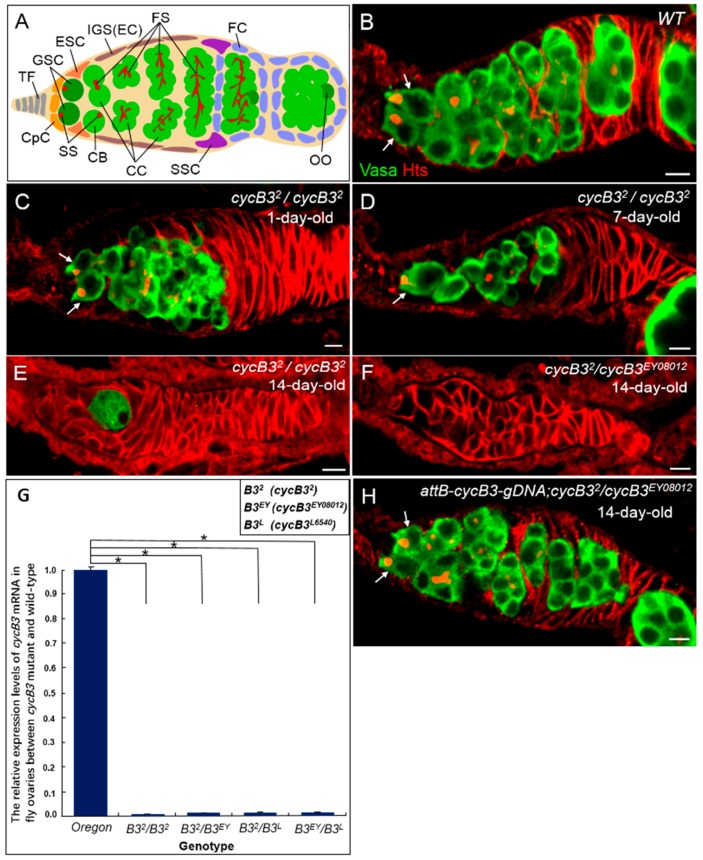
*cycB3* is required for maintaining GSCs in the *Drosophila* ovary. (**A**) A cross-sectional diagram of an adult *Drosophila* germarium; (**B**–**F**,**H**) germaria labeled with anti-Vasa antibody (green, germ cells), and with anti-Hts antibody (red, fusomes and spectrosomes). GSCs are indicated by arrows. (**B**) Wild-type germarium with two GSCs; (**C**–**F**) *cycB3* mutant ovaries on different days after eclosion. Germaria, containing two GSCs (**C**); one GSC (**D**); no GSCs (**E**) and an empty germarium (**F**); (**G**) Quantitative real-time PCR analyses of *cycB3* mRNA levels in ovaries between wild-type and *cycB3* mutants; (**H**) The transgene P{*attB*-*cycB3-gDNA*} rescued the *cycB3*^2^/*cycB3^EY^*^08012^ mutant ovary to normal. Scale bars: 5 μm. * *p* < 0.001. Abbreviations: Terminal filament (TF), Germline stem cell (GSC), Escort stem cell (ESC), Inner germarium sheath cell (IGS) or Escort cell (EC), Fusomes (FS), Follicle cell (FC), Cap cell (CpC), Spectrosomes (SS), Cystoblast (CB), Cyst cell (CC), Somatic stem cell (SSC), OO(oocyte).

Adult stem cells are characterized by their ability to supply new cells to replace aged/injured cells in adult tissues throughout life, and to maintain their “stemness”, via self-renewal. It is essential for stem cells to keep a balance between self-renewal and differentiation into daughter cells. Numerous studies over the past twenty years have shown that the stem cell maintenance is modulated by intrinsic and extrinsic mechanisms [1,2]. Germline stem cells (GSCs) in the *Drosophila* ovary provide an excellent model for exploring the mechanisms underlying GSC fate determination, in vivo. The *Drosophila* adult ovary contains about fifteen ovarioles, in which an anatomical structure, called germarium, is positioned at the apical end. Two to three GSCs are located in the anterior region of the germarium, and three types of somatic cells (terminal filament cells, cap cells and escort stem cells) constitute the microenvironment (also called the “niche”) for GSCs (Figure 1A) [3,4]. A GSC divides asymmetrically to give birth to two daughters—one daughter cell remains adherent to niche cells and continuously functions as a stem cell, whereas the other daughter moves away from the niche and initiates differentiation as a cystoblast (CB). GSCs are readily visualized by a spherical spectrosome, which is located in the anterior region in the cell, while the spectrosome in CB usually loses its anterior localization. CB undergoes four rounds of successive incomplete mitosis and generates a 16-cell germ line cyst, interconnected by a branched fusome. The 16-cell cyst is surrounded by follicle cells, derived from the somatic stem cell (SSC), then the encapsulated cyst moves posteriorly out of the germarium and forms the egg chamber, eventually developing into a mature egg.

Previous research has manifested that Bmp/Dpp-bam functions as the primary signaling pathway for GSC maintenance in the *Drosophila* ovary [5,6,7,8,9]. Bmp, produced by niche cells, acts as a short-range signal, which eventually represses *bam* transcription in GSCs, to maintain its self-renewal [10,11]. Ectopic overexpression of Bmp in cap cells can suppress CB differentiation and produce an ovarian tumor, whereas the reduced level of Bmps directly results in the loss of the GSC phenotype [6,7,8,9,10,11]. In addition to the Bmp-dependent extrinsic regulatory mechanism, the fate of GSCs is also controlled by intrinsic regulatory factors, such as Nanos, Pumillio, Cyclin B, Cyclin E, Ote and Effete and Gcn5 [12,13,14,15,16,17,18]. Even so, many extrinsic/intrinsic regulatory factors from niche cells/GSCs remain to be identified.

Cyclin proteins are characterized by their periodical appearance, accumulation and degradation during cell-cycle progression. Since the first *cyclin* (*cyc*), *cycA*, was cloned and found with a periodic expression pattern in cell-cycle [19,20], many other types of *cyclins* (e.g., *cyclin B*, *C*, *D*, *E* and *K*) were subsequently found [21,22,23,24,25]. Cyclins are positive regulatory subunits of Cyclin-dependent kinases (CDKs), whereby CDKs play a key role in the control of cell-cycle transitions. Studies in numerous organisms have demonstrated that at least three evolutionarily-conserved classes of mitotic *cyclins*, *cycA*, *B* and *B3*, have overlapping, but nonidentical, functions for mitosis progressions (i.e., prophase, metaphase, anaphase and telophase) [26,27,28,29,30]. In addition, ubiquitin-mediated sequential degradation of these Cyclins is also essential for dividing cells to exit mitosis, leading to eventual completion of cell-cycle [26,27,30]. Cyclins display regulatory functions in controlling the fate of stem cells (self-renewal or switch to differentiation). For example, the expression level of CycA is involved in maintaining the fate of GSCs in the *Drosophila* ovary [9,15,18]. *cycB* is required for GSC maintenance in the female *Drosophila* [16]. Knockout of *cycD3* leads to impaired establishment of the skeletal muscle satellite cell (i.e., muscle stem cell) population within adult mouse muscle tissues [31]. Higher Cyclin E-cdk2 kinase activity is required for ovarian follicle stem cell maintenance [32]. Cyclin H plays a critical role in maintaining ESC (embryonic stem cell) identity [33]. Cyclin K protein exhibits a high expression level in pluripotent embryonic stem cells but low in their differentiated derivatives or tissue-specific stem cells, and knockdown of *cyclin K* leads to cell differentiation [34]. Here, we have uncovered a new role for *cycB3* that plays a key role in maintaining the fate of GSCs in the *Drosophila* ovary.

## 2. Results

### 2.1. Deficiency of cycB3 Impairs GSCs Maintenance in Drosophila Ovary

To discover the genes that possibly influence the fate of *Drosophila* GSCs, we performed a genetic screen of female sterile lines. We isolated a null allele, *cycB3*^2^, which carries a small deletion resulting from an imprecise excision of the P-element insertion in *cyclin B3* [29]. Quite a few *cycB3*^2^ homozygous mutant flies (>30%) displayed slim ovaries when dissected at day 7 after eclosion. This finding triggered us to thoroughly examine the behavior of GSCs in *cycB3* mutant. We first obtained two additional P-element insertion mutation alleles of the gene *cycB3* (i.e., P{EPgy2} *cycB3^EY^*^08012^ and P{lacW} *cycB3^L^*^6540^) from Bloomington Stock Center [35]. Next, we got three trans-heterozygous mutants (i.e., *cycB3*^2^/*cycB3^EY^*^08012^, *cycB3*^2^/*cycB3^L^*^6540^ and *cycB3^EY^*^08012^/*cycB3^L^*^6540^) other than the *cycB3*^2^ homozygous mutant (*cycB3*^2^/*cycB3*^2^). We used anti-Hts and anti-Vasa antibodies to visualize the spectrosomes/fusomes and germ cells in adult ovaries, respectively (Figure 1B). Vasa displays a germ cell-specific expression pattern, while Hts exhibits an enrichment in both round fusomes (i.e., spectrosomes) and branched fusomes [36,37]. By using the methods described previously [15], we finally performed phenotypic analyses by measuring the ratios of four types of germaria (i.e., 2–3 GSCs-, 1 GSC-, 0 GSC-containing and empty germaria) in different *cycB3* mutation backgrounds, at different ages.

As shown in Table 1, in wild-type ovaries, the number of normal germaria (containing 2–3 GSCs), examined at three stages (days 1, 7 and 14, post-eclosion) was largely sustained at high levels, measured as 98.6% (*n* = 146), 95.5% (*n* = 178) and 90.5% (*n* = 148), respectively (Figure 1B). In contrast, the number of normal germaria from *cycB3*^2^ homozygotes at three stages (day 1, 7 and 14) was reduced dramatically with time, counted as 89.1% (*n* = 274), 39.4% (*n* = 203) and 22.6% (*n* = 420), respectively (Table 1 and Figure 1C). Contrarily, the ratios of abnormal phenotypes (1 GSC, 0 GSC and empty germaria) from *cycB3*^2^ homozygote were increased from the initial 10.9% (30/274), at day 1, to the final 77.4% (325/420), at day 14 (Table 1 and Figure 1D,E). These data demonstrate that the number of abnormal germaria in *cycB3*^2^ mutant ovaries was elevated rapidly as time elapsed, suggesting a notable loss of GSCs in *cycB3* deficient ovaries. Similarly, the ratios of normal germaria from three *cycB3* trans-heterozygotes (*cycB3*^2^/*cycB3^EY^*^08012^, *cycB3*^2^/*cycB3^L^*^6540^ and *cycB3^EY^*^08012^/*cycB3^L^*^6540^), at day 1, were 94.3% (*n* = 209), 86.6% (*n* = 194) and 80.8% (*n* = 219), respectively (Table 1). Two weeks later, the ratios of normal germaria had decreased severely, measured as 27.6% (*n* = 340), 28.2% (*n* = 444) and 18.4% (*n* = 207), respectively (Table 1). Accordingly, the numbers of abnormal germaria from three *cycB3* trans-heterozygotes increased with time. In these abnormal phenotypes, the total proportions of 0 GSC and empty germaria, from three trans-heterozygotes, at day 14, were increased to 44.4% (151/340), 45.3% (201/444) and 48.7% (101/207), respectively (Table 1 and Figure 1F). In addition, similar results were observed in *cycB3*^2^*/Df* (fly deficiency strain of *cycB3* gene) mutant ovaries (Table 1). Taken together, these statistical data strongly suggest that *cycB3* deficiency causes a progressive loss of GSCs with ageing.

To determine if the GSC loss phenotype in *cycB3* mutant ovaries is due to a reduced *cycB3* expression level, we performed quantitative real-time polymerase chain reaction (qPCR) assays, to compare mRNA levels between wild-type and mutant ovaries [38]. According to the method described previously [39], we extracted total RNA from *Drosophila* ovaries and performed reverse transcription-based qPCR experiments to measure the *cycB3* mRNA level, using the *rp49* gene as a reference. Compared with wild-types, the *cycB3* mRNA expression level in *cycB3* mutant ovaries (*cycB3*^2^/*cycB3*^2^, *cycB3*^2^/*cycB3^EY^*^08012^, *cycB3*^2^/*cycB3^L^*^6540^ and *cycB3^EY^*^08012^/*cycB3^L^*^6540^) was reduced dramatically (Figure 1G). The data strongly suggest that *cycB3* is reduced in *cycB3* mutant ovaries, indicating that *cycB3* protein is responsible for the loss of GSC phenotypes in *cycB3* mutant flies.

To confirm a specific role of *cycB3* in GSC maintenance, we performed a *cycB3* rescue assay, by generating a transgene of P{*attB*-*cycB3-gDNA*}, in which a 9.5 kb genomic DNA fragment, encompassing the ~2.8 kb *cycB3* transcript, was introduced into *attP*-*phiC31* fly hosts, by *attB/attP*-element-mediated germline transformation [40]. We found that the GSC loss phenotype in three *cycB3* allelic mutants was fully rescued by this transgenic line (Figure 1H and Table S1). Taken together, these results substantiate the idea that *cycB3* plays an essential role in GSC maintenance.

### 2.2. The Gene cycB3 Functions as an Intrinsic Factor in Controlling GSC Maintenance

**Figure 2:**
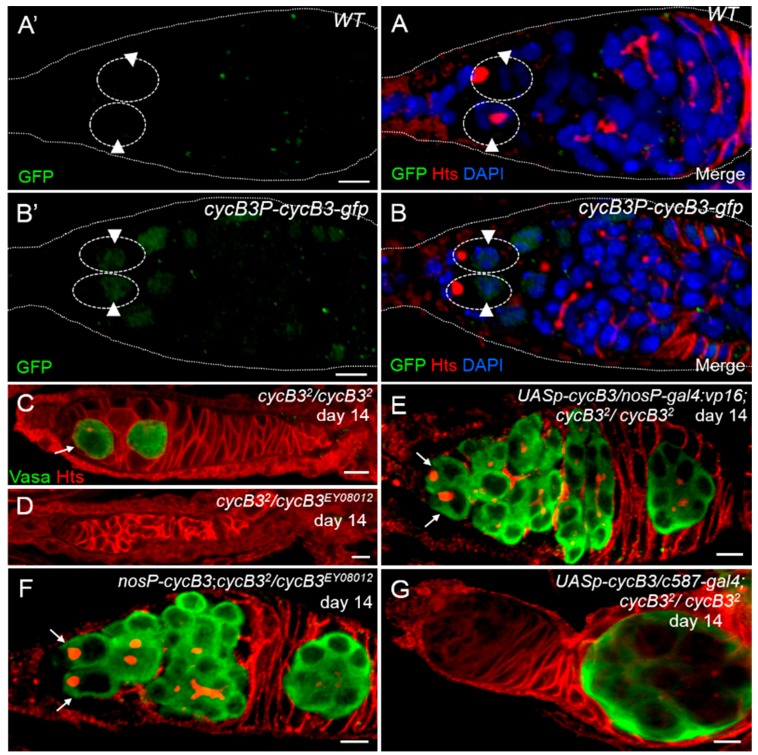
*cycB3* is required intrinsically for controlling the fate of GSCs. (**A**,**B**) Ovaries were stained with anti-Hts antibody (red) to visualize fusomes, 4',6-diamidino-2-phenylindole (DAPI) dye to label nuclei (blue), and anti-GFP antibody (green) to show the *cycB3* expression pattern; (**A’**,**A**) Ovary from an *Oregon* fly (wild-type). GSCs (indicated by dashed circles) were GFP negative (noted by arrowheads); (**B’**,**B**) Ovary carrying a transgene P{*cycB3P-cycB3-gfp*}. GSCs (indicated by dashed circles) were GFP positive (green, noted by arrowheads); (**C**–**G**) Fourteen-day-old germaria stained with anti-Vasa antibody (green, germ cells), and with anti-Hts antibody (red, spectrosomes and fusomes). GSCs are indicated by arrows. (**C**) *cycB3* mutant germarium with one GSC; (**D**) *cycB3* mutant with empty germarium. (**E**,**F**) *cycB3* mutant ovaries were rescued by the transgenes of *nos-gal4:vp16*; *UASp-cycB3* and *nosP-cycB3*. Germarium with two GSCs. (**G**) *cycB3* mutant flies, carrying the genotype, *c587-gal4*; *UASp-cycB3*. Ovary with an empty germarium. Scale bars: 5 μm.

It has been reported that the GSC maintenance is modulated by intrinsic and extrinsic signaling pathways in the ovary [1,5,10,13]. To determine the role of *cycB3* in the GSC maintenance, we generated a transgenic reporter, P{*cycB3P-cycB3-gfp*}, in which the *cycB3-gfp* fused coding sequence (encoding CycB3-Green fluorescent protein (GFP) fusion protein) was placed under the control of a 6.5 kb *cycB3* promoter. The *gfp* expression pattern faithfully reflects *cycB3* gene expression in this reporter system [41]. Thus, GFP expression can be used to represent that of *cycB3*. GFP expression was checked in transgenic fly ovaries with wild-types as a control (Figure 2A’,A). We found that GFP showed a specific expression in germ cells (e.g., GSCs) of fly ovaries (*n* > 100 germaria), and GFP was localized to the cell nuclei of GSCs (Figure 2B’,B), which is consistent with previous studies [29,42]. Therefore, this result suggests that *cycB3* functions as an intrinsic factor.

To further test its intrinsic role, we performed tissue-specific rescue assays, under a *cycB3* mutant background, by using the Gal4-*UAS* system [43], in which *cycB3* protein could be expressed specifically, either in germ cells (e.g., GSCs) or in somatic cells (e.g., niche cells). When doing rescue assays, all of the tested flies were raised at 29 °C after eclosion, to obtain a higher activity of Gal4, which can enhance phenotypic severity [44]. We first generated a transgene, P{*UASp-cycB3*}, in which the *cycB3* coding sequence was under the control of the *UASp* promoter [15]. Then we forcibly expressed this transgene by either the extrinsic or intrinsic driver (i.e., *c587-gal4* and *nanosP-gal4:vp16*). We found that, compared to *cycB3* mutants, the GSC loss phenotype was fully rescued in *cycB3* mutant ovaries carrying the transgenes of P{*UASp-cycB3*} and P{*nanosP-gal4:vp16*} (abbreviated as P{*nosP-gvp*} in Figure 2) (*p* < 0.001, *χ*^2^ test), in which the *cycB3* protein was specifically/highly expressed by a germline-specific *nosP-gvp* driver [45,46] (Figure 2C–E and Table S2). This result demonstrates that the intrinsic supplement of *cycB3* completely rescues the GSC loss phenotype in *cycB3* mutant ovaries, indicating that *cycB3* intrinsically plays a role in GSC maintenance. To confirm this result, we generated another transgene, P{*nosP-cycB3*}, in which the *cycB3* coding sequence was placed under the control of the promoter of the *nanos* gene, which exhibits a high expression level in germline cells. We observed that the GSC loss phenotype was fully rescued under a *cycB3* mutant background (Figure 2F and Table S2). To exclude the possibility that *cycB3* also plays an extrinsic role in GSC maintenance, we extrinsically expressed *cycB3* protein in somatic niche cells by *c587-gal4*-driven *UASp*-*cycB3* expression [15]. We found that the GSC loss phenotype was not rescued in *cycB3* mutant ovaries carrying the *UASp*-*cycB3* and *c587-gal4* transgenes (Figure 2G and Table S2). Taken together, the data demonstrate that *cycB3* is required intrinsically for GSC maintenance.

**Figure 3:**
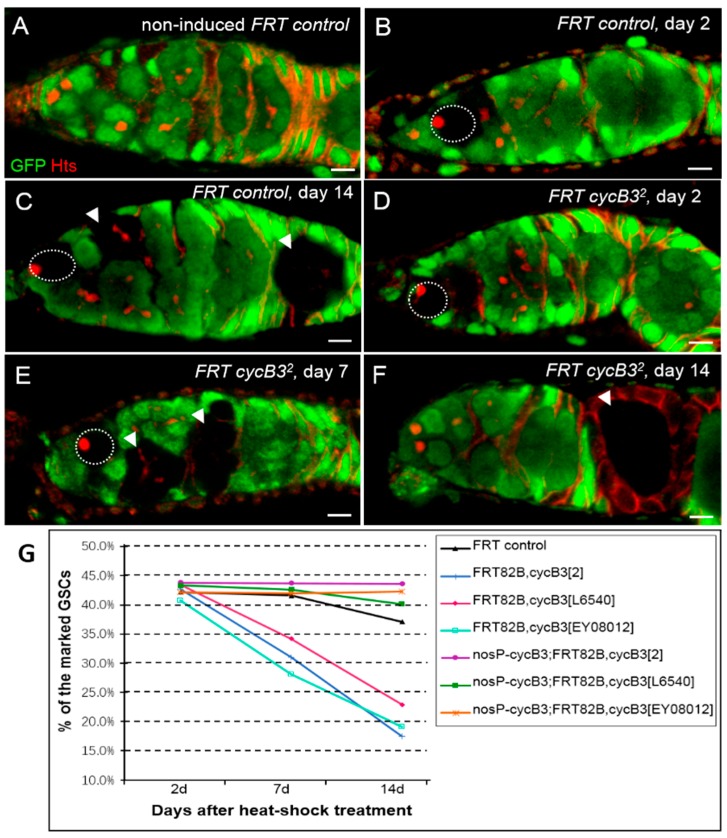
The intrinsic deficiency of *cycB3* leads to GSC loss in the *Drosophila* ovary. (**A**–**F**) Ovarioles from *FRT* control (**A**–**C**) and *FRT*, *cycB3* flies (**D**–**F**) were collected at different days after heat-shock treatment and stained with anti-GFP (green) and anti-Hts (red) antibodies. GFP-negatively marked GSC clones (indicated by dashed circles) and cyst clones (noted by arrowheads); (**G**) Percentages of negatively GFP-marked GSC clones in *FRT* control and *cycB3* mutant alleles, at days 2, 7 and 14. Compared with marked GSCs (GFP-) in *FRT* control, the percentages of marked *cycB3* mutant GSCs (GFP-) decreased dramatically. Scale bars: 5 μm.

To further determine whether the intrinsic deficiency of *cycB3* in GSCs is sufficient to result in the GSC loss phenotype, we used the FLP (flipase) /*FRT*-mediated mitotic recombination technique to generate marked *cycB3* mutant GSC clones [14,47]. We analyzed the loss rate of GFP-negatively marked GSC clones, according to the method described previously [14,15]. The *cycB3* mutant GSCs were marked by the absence of GFP expression after five days of heat-shock treatment. Using this system, we examined the loss rates of the marked GSCs between *FRT* control (*hs-flp/+*; *FRT82B/FRT82B*) and *cycB3* mutant flies (*hs-flp/+*; *FRT82B*, *cycB3/FRT82B*, *cycB3*), at days 2, 7 and 14 after heat-shock treatments (AHST). In the non-heat-shock *FRT* control, GFP was expressed ubiquitously in *Drosophila* ovary (Figure 3A). For the *FRT* control, the percentages of marked GSC clones reduced weakly, from the initial 42.3% (*n* = 130) to the final 37.3% (*n* = 142), during a period of 12 days (Figure 3B,C,G). The data showed that merely 11.8% of the marked GSCs were lost during the 12-day AHST period. In contrast, the rates of marked *cycB3* mutant GSC clones (*cycB3*^2^, *cycB3^EY^*^08012^ and *cycB3^L^*^6540^) decreased rapidly from the initial 42.4% (*n* = 158), 40.9% (*n* = 132) and 43.6% (*n* = 163), respectively, at day 2 AHST, to the final 17.2% (*n* = 209), 19.2% (*n* = 151) and 23.0% (*n* = 152), respectively, at day 14 AHST (Figure 3D–F,G). These results indicated that 59.0%, 53.1% and 47.5% of marked *cycB3*^2^, *cycB3^EY^*^08012^ and *cycB3^L^*^6540^ mutant GSCs were lost during the measured 12-day AHST. Put together, these findings suggest that *cycB3* plays an intrinsic role for GSC maintenance.

To substantiate the role of *cycB3* as an intrinsic GSC maintenance regulator, we performed a rescue assay in *cycB3* mutant GSC clones, by supplementing *cycB3* function, using the transgene of P{*nosP-cycB3*} [15]. As shown in Figure 3G, the rates of marked *cycB3* mutant GSC clones (*cycB3*^2^, *cycB3^EY^*^08012^ and *cycB3^L^*^6540^) decreased very weakly, from the initial 43.7% (*n* = 151), 42.6% (*n* = 122) and 43.0% (*n* = 149), respectively, at day 2 AHST, to the final 43.6% (*n* = 172), 42.2% (*n* = 161) and 40.1% (*n* = 192), respectively at day 14 AHST. The data showed that only 0.2%, 0.9% and 6.7% of marked *cycB3*^2^, *cycB3^EY0^*^8012^ and *cycB3^L^*^6540^ mutant GSCs were lost during the testing days. There were no differences between the *FRT* control and each of the rescue alleles (*p* > 0.05, *χ*^2^ test). Taken together, these results strongly support that idea that *cycB3* functions as an intrinsic modulator for GSC maintenance.

### 2.3. cycB3 Does Not Influence Bmp/dpp-Mediated Dad Expression

**Figure 4:**
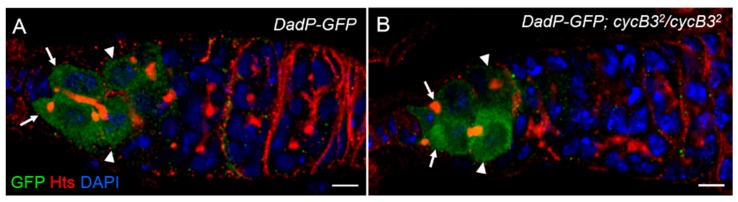
*cycB3* doesn’t affect *Dad* expression pattern. (**A**,**B**) Ovaries were stained with anti-GFP (green), anti-Hts (red) antibodies and DAPI (blue). GSCs are indicated by arrows, CBs are noted by arrowheads. Germaria from both *Dad-GFP* (**A**) and *Dad-GFP*; *cycB3*^2^/*cycB3*^2^ (**B**) exhibited a higher GFP expression level in GSCs, and a lower level in CBs. Five-day-old flies were analyzed. Scale bars: 5 μm.

The Bmp/Dpp target gene, *Daughters against Dpp* (*Dad*), whose expression is induced by Dpp, negatively regulates Dpp signaling and forms a negative-feedback loop in *Drosophila* wing development [48,49]. To explore whether *cycB3* affects Dpp signaling, we examined the *Dad* expression pattern in the *cycB3* mutant background with the transgenic reporter, *DadP-GFP*, in which the *GFP*-coding sequence was positioned downstream of the promoter of the gene *Dad*, so that the GFP expression pattern represented that of *Dad* [50]. As shown in Figure 4A, the *DadP-GFP* (designed as wild-type control) expression within the germline is strong in GSCs, which is rapidly downregulated in CBs from the 5-day-old ovaries (*n* > 150 germaria) [5,51]. A similar *Dad* expression pattern was also found in the *cycB3* mutants (*DadP-GFP*; *cycB3*^2^/*cycB3*^2^) ovaries (*n* > 250 germaria) (Figure 4B). These results suggest that *cycB3* is not involved in *Dad*-mediated regulation of Bmp signaling.

### 2.4. cycB3 Is Not Required for Bam Transcriptional Silencing and Acts Probably in a Bam-Dependent Manner, in Ovarian GSCs of Drosophila

**Figure 5:**
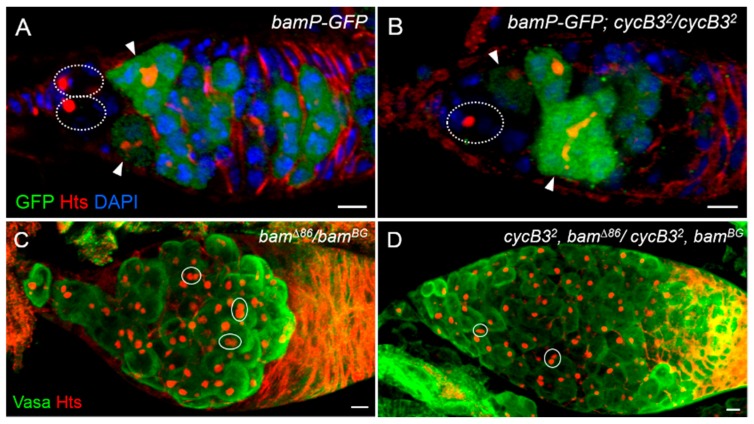
The genetic relationship between *cycB3* and *bam*. (**A**,**B**) *cycB3* is not required for *bam* transcriptional silencing. Ovaries were stained with anti-GFP (green), anti-Hts (red) antibodies and DAPI (blue). GSCs are indicated by dashed circles. CBs and cyst cells are noted by arrowheads. Germaria both from *bamP-GFP* (**A**) and *bamP-GFP*; *cycB3*^2^/*cycB3*^2^ (**B**) exhibited a *bam-GFP* negative expression in GSCs, and a *bam-GFP* positive expression in CBs and cyst cells; (**C**,**D**) The gene *bam* mutation suppresses the GSC loss caused by a *cycB3* deficit. Ovaries were stained with anti-Vasa (green) and anti-Hts (red) antibodies. Germaria both from *bam* single mutants (**C**) and *bam-cycB3* double mutants (**D**) possessed a lot of GSC-like cells, carrying either round spectrosomes or associated round spectrosomes (noted by solid circles). Scale bars: 5 μm.

It is known that Dpp-dependent *bam* transcriptional silencing is an essential mechanism for GSC self-renewal [5,10,11]. To test if *cycB3* is involved in *bam* silencing, we examined *bam* expression patterns in *cycB3* mutant ovaries, by observing the *GFP* expression with the *bam* transcriptional reporter, P{*bamP-GFP*} (a *GFP* coding sequence driven by a *bam* promoter) [10,15]. We found that, by marking the germ cells derived from 5-day-old flies with two antibodies (anti-Hts and anti-GFP) and 4′,6-diamidino-2-phenylindole (DAPI) staining, all of the putative ovarian GSCs from wild-type and *cycB3*^2^ (a loss of function allele) [29] homozygote exhibited a negative GFP pattern (wild-type, *n* > 150 germaria; *cycB3* mutant, *n* > 200 germaria) (Figure 5A,B). These results suggest that *cycB3* is dispensable for *bam* silencing. Thus, *cycB3* function is not located upstream of *bam* action in ovarian GSCs of *Drosophila*.

To determine whether the *cycB3* gene functions in a *bam*-independent manner, we generated *cycB3* and *bam* double mutant flies (*cycB3*^2^, *bam^Δ^*^86^/*cycB3*^2^, *bam^BG^*). In *bam-*single mutant ovaries from 14-day-old flies, we found that all germaria contained non-differentiated germ cells with characteristics similar to GSCs (GSC-like cells carrying either round spectrosomes or associated round spectrosomes) (Figure 5C) [10]. As shown in Figure 5D, the *cycB3*; *bam*-double mutants phenocopied the *bam* single mutants, by producing morphological germarium tumors (*n* > 150 germaria). The data demonstrate that the deficiency of *bam* represses the loss of GSCs caused by *cycB3* mutations in the *Drosophila* ovary, suggesting that *bam+* activity is required for *cycB3* mutant GSC differentiation. Thus, we propose that *cycB3* functions probably in a *bam*-dependent manner.

### 2.5. Deficiency of cycB3 Fails to Cause Apoptosis in Ovarian GSCs, Nor Influences CB Differentiation into Oocytes in Drosophila Ovary

**Figure 6:**
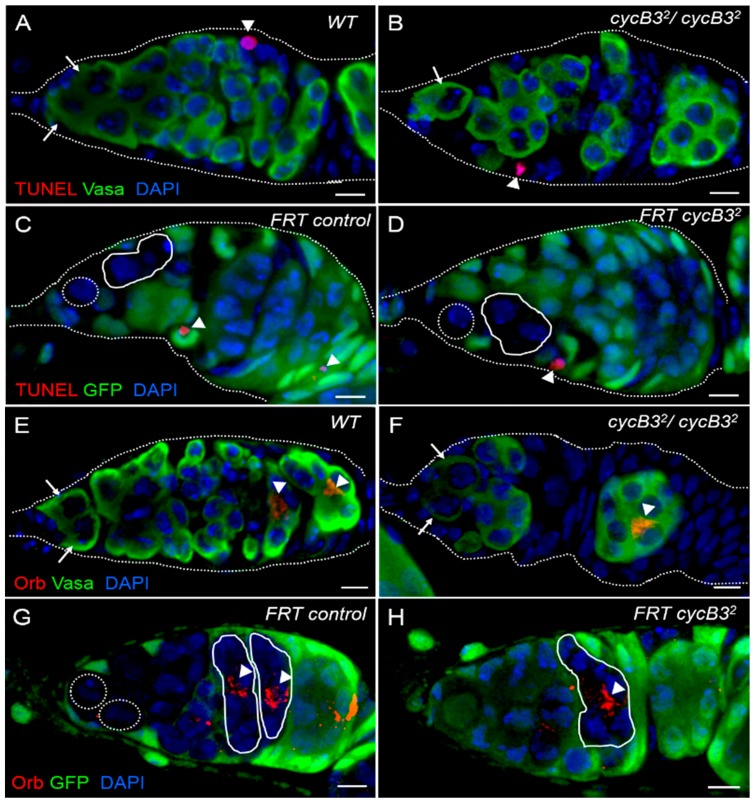
The mutation in *cycB3* fails to affect oocyte formation. (**A**–**D**) The deficiency of *cycB3* fails to lead to apoptosis in GSCs. Germaria from wild-type (**A**) and *cycB3*^2^ mutants (**B**) were labeled by TUNEL (red, indicated by arrowheads) and stained with anti-Vasa antibody (green) and DAPI dye (blue); Germaria from *FRT* controls (**C**) and *FRT cycB3*^2^ mutants (**D**) were labeled by TUNEL (red, indicated by arrowheads), stained with anti-GFP antibody (green) and DAPI (blue); (**E**–**H**) The *cycB3* mutation in CB fails to impair its differentiation into oocytes; Germaria from wild-type (**E**) and *cycB3*^2^ mutants (**F**) were stained with anti-Orb antibody (red, indicated by arrowheads), anti-Vasa antibody (green) and DAPI (blue). The outlines of germaria are drawn by dashed circles. Germaria from *FRT* controls (**G**) and *FRT cycB3*^2^ mutants (**H**) were stained by anti-Orb antibody (red, indicated by arrowheads), anti-GFP antibody (green) and DAPI (blue). GSCs are noted by arrows (**A**,**B**,**E**,**F**); GSC clones are indicated by dashed circles and cyst clones are noted by solid circles (**C**,**D**,**G**,**H**). The outlines of germaria are drawn with dashed circles (**A**–**F**). Scale bars: 5 μm.

To explore whether the loss of GSCs in *cycB3* mutants was caused by its apoptosis-mediated cell death, we examined the rate of apoptosis in *cycB3* mutant GSCs with Terminal deoxynucleotidyl transferase-mediated dUTP Nick End Labeling (TUNEL) assays [15]. We found that the apoptosis-occurring rates, in wild-type and *cycB3*^2^ null mutant ovaries, at day 4 after eclosion, were 1.4% (3/210) and 1.8% (4/223), respectively (Figure 6A,B and Table S3), suggesting that there are no enhanced apoptosis rates in *cycB3*^2^ null mutant GSCs. Similar observations were also found in FLP-FRT-induced *cycB3*^2^ mutant GSC clones (i.e., GFP negatively-marked GSCs) in *Drosophila* ovaries. The apoptosis-occurring rates at day 6 AHST in *FRT* control and marked *cycB3* mutant GSCs were 1.5% (3/201) and 1.0% (2/197), respectively (Figure 6C,D and Table S3). Put together, these results showed that the *cycB3* mutation failed to cause apoptosis in ovarian GSCs, suggesting that mutant GSCs may precociously differentiate into CBs.

CBs can develop into cyst cells, eventually differentiate into oocytes during *Drosophila* oogenesis. If this process is disturbed, oocyte formation will be blocked. To determine whether the oocytes form normally in *cycB3* mutant ovaries, we examined the expression of *orb* in *cycB3* mutant ovaries. Orb protein preferentially accumulates in the newly formed oocytes [52], therefore Orb can be used as a marker of oocytes, to show the status of CB differentiation [53]. We found that the oocytes from both wild-type (*n* = 150 germaria) and *cycB3*^2^ null mutants (*n* = 165 germaria) showed normal Orb-positive expression patterns in germaria (Figure 6E,F). Similar results were also observed in *cycB3* mutant germaria, induced by FLP/*FRT*-mediated mitotic recombination. All the oocytes located in the 16-cell cyst-clones from *FRT* controls (*n* = 80 cyst clones) and *cycB3*^2^ mutants (*n* = 100) exhibited normal Orb-positive staining (Figure 6G,H). Taken together, these data strongly indicate that *cycB3* has no effects on CB differentiation.

### 2.6. Over-Expression of cycB3 Fails to Increases the Number of GSCs, but Dramatically Enhances the Number of CBs

Since a loss of function of *cycB3* resulted in a loss of ovarian GSCs, while no enhanced apoptosis rates were measured in *cycB3* mutant female GSCs, we hypothesized that an excess of *cycB3* could promote GSC proliferation or/and delay CB differentiation. To test this hypothesis, we first generated the transgenic fly lines that carried the transgenes of P{*attB*-*cycB3-gDNA*} and P{*nosP-cycB3*}, as described in the above paragraph. Then, these two transgenic lines were crossed with the allele, P{*bamP-GFP*}, a *bam* transcriptional reporter [10]. Finally, we obtained two *cycB3*-overexpression strains, *bamP-GFP*; *attB*-*cycB3-gDNA* and *bamP-GFP*; *nosP-cycB3*, in which GSCs and CBs were clearly recognized by immunostaining anti-GFP and anti-Hts antibodies [10,36]. Fusomes are morphologically spherical spectrosomes in GSC/CBs (or a connected short bar in the case of a dividing GSC/CB) and branched in differentiated cysts (Figure 1A,B). GSCs and CBs are known to have negative and positive Bam-staining patterns, respectively [10,14].

**Figure 7:**
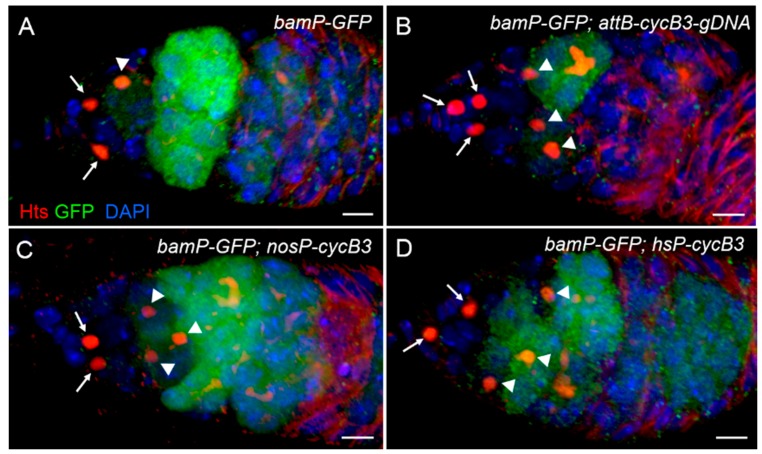
Over-expression of *cycB3* notably increases the number of CBs. Ovaries from wild-type (**A**) and *cycB3*-overexpression flies (**B**–**D**) were stained with anti-Hts antibody (red), anti-Vasa antibody (green), and DAPI dye (blue). The micrographs were stacked together in the Z-axis direction, to visualize spectrosome-containing GSCs (indicated by arrows) and CBs (noted by arrowheads). Scale bars: 5 μm.

We measured the number of germ cells carrying spectrosomes in germaria from wild-type (i.e., *bamP-GFP*) and *cycB3*-overexpressed flies, at day 10 after eclosion. As shown in Table 2, in wild-types, the average numbers of spectrosome-containing GSCs and CBs (SGAC) were 2.1 and 1.1 per germarium (*n* = 87), respectively (Figure 7A). In contrast, the numbers of SGAC from *cycB3*-overexpressed flies carrying *bamP-GFP*; *attB*-*cycB3-gDNA* were 2.6 and 2.8 per germarium (*n* = 83), respectively (Figure 7B and Table 2). These results demonstrated that, compared to wild-types, there was no obvious increase in GSC number (*p* < 0.05), but a dramatically enhanced CB number was observed (*p* < 0.01). Similar results were also observed in *cycB3*-overexpression ovaries of *bamP-GFP*; *nosP-cycB3*, and the average numbers of SGAC were 3.0 and 3.1 per germarium (*n* = 113), respectively (Figure 7C and Table 2).

To confirm these results, we generated a new transgenic line, carrying P{*hsP-cycB3*}, in which the *cycB3*-coding sequence was placed downstream of the heat-shock promoter [54]. The *cycB3* overexpression in ovaries was stimulated by heat-shock, at 37 °C, for 60 min each time, for a total of three times per day. After seven consecutive days of heat-shock treatment, we measured the average numbers of SGAC, with P{*hsP-cycB3*} flies raised at 25 °C as a control. We found that there was no difference in the spectrosome-containing GSC number between the control and heat-shock flies (*p* > 0.05) (Figure 7D and Table 2). However, the average number of CBs was notably increased to 3.2 per germarium (*n* = 98), compared with the control flies (1.1 per germarium, *n* = 89), cultured at room temperature (*p* < 0.01). Taken together, these data suggest that enhanced *cycB3* activity efficiently suppresses CB differentiation, but is not sufficient to accelerate GSC proliferation.

## 3. Discussion

The *cycB3* gene is evolutionarily-conserved among higher eukaryotic organisms examined, from insects to mammalians [27,29,42,55,56]. The *cycB3* protein is present as Cyclin A and B (two other B-type Cyclins) in mitotically-proliferating cells, and is involved in the regulation of mitosis, where it cooperates with Cyclin A and B [29,42]. It is reported that Cyclin A and B are involved in the regulation of ovarian GSC maintenance in *Drosophila* [9,15,16,18]. Our earlier observation showed that *cycB3*^2^ homozygous mutant females partially exhibit thinned ovaries. Given the above reports on Cyclin activity in stem cells, the thinned ovaries prompted us to further explore the potential involvement of *cycB3* in the maintenance of germline stem cells, in the *Drosophila* ovary. The phenotypic assays indicate that a *cycB3* deficiency leads to GSC loss with ageing. The rescue assays and genetic mosaic analyses convincingly suggest that *cycB3* functions as an intrinsic factor for controlling the fate of GSC.

**Figure 8:**
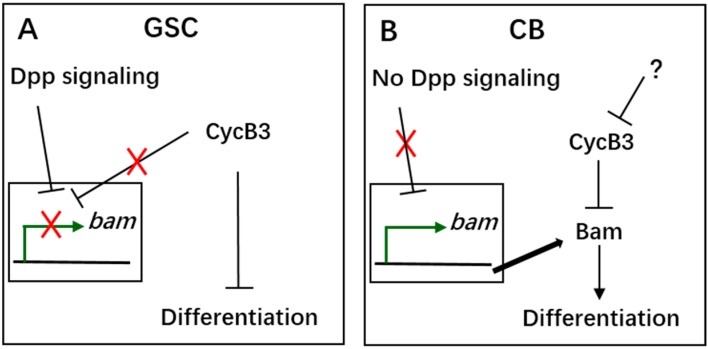
Model to explain how *cycB3* regulates GSC self-renewal and CB differentiation. (**A**) *cycB3* is required for GSC maintenance, because it represses GSC differentiation; (**B**) Bam is necessary for CB differentiation and *cycB3* function is positioned upstream of Bam action in CBs.

Previous studies have discovered that the *dpp/bam* pathway is the essential signaling pathway for maintaining GSCs in the *Drosophila* ovary [5,6,7,8,9,10,11]. The *bam* gene is a key switch in regulating the fate of GSC [11]. Here, combining our results, we proposed a model to explain how *cycB3* is involved in regulation of GSC/CB fate determination (Figure 8). In GSCs, our data show that *cycB3* is not involved in Dpp-mediated *bam* transcriptional silencing (Figure 8A). The *cycB3* deficiency triggers GSC pre-differentiation and eventually causes its loss phenotype. In CBs, the *bam* gene exhibits a high expression level, due to loss of the inhibition by Dpp signaling (Figure 5A), and the Bam protein can promote CB differentiation [10,11]. As shown in Figure 8B, our genetic interaction analyses strongly show that *cycB3* function is positioned upstream of Bam action in CBs. The excess *cycB3* come from *cycB3* overexpression, which specifically suppresses CB differentiation, probably through repressing the activity of Bam. However, what are the factors that functionally position upstream of *cycB3* in CBs of *Drosophila* ovary? This still remains elusive.

It is reported that *cycB3* promotes metaphase–anaphase transition in *Drosophila* embryos [57]. Our data show that overexpression of *cycB3* fails to increase the number of GSCs, suggesting that the excess *cycB3* may fail to influence transition into the GSC system, whereas the excess *cycB3* is sufficient to delay CB differentiation. The underlying molecular mechanism might be due to the fact that the increased *cycB3* activity is sufficient to enhance CB proliferation, by promoting metaphase-anaphase transition.

## 4. Materials and Methods

### 4.1. Constructs

The *pattB*-*UASp*, *pattB*-*nosP* and *pattB*-*hsP* vectors (abbreviated as *UASp*, *nosP* and *hsP*) were constructed according to a previous method [58]. To generate the *attB*-*cycB3-gDNA* construct, the genomic DNA (gDNA) was prepared from wild-type flies, as described previously [59], which was used as template in PCR reactions, to amplify the 9.5 kb length of the *cycB3* gDNA fragment (P1/P2 as primers, Table S4). Then, this fragment was subcloned to *nosP* with the restriction enzymes, *SbfI* and *KpnI*. To make the *UASp-cycB3*, *hsP-cycB3* and *nosP-cycB3* constructs, total RNA was isolated from wild-type ovaries and reverse-transcription was performed, using the methods and reagents described previously [59]. Then the total ovarian cDNA was used as a template to amplify the *cycB3*-coding sequence (P3/P4 as primers, Table S4), which was subcloned to *UASp*, *nosP* and *hsP*, with *AscI* and *kpnI.* To make the *cycB3P-cycB3-gfp* construct, the *attB*-*cycB3-gDNA* construct was used as a template to amplify the 6.5 kb length of the *cycB3* promoter (P1/P5 as primers, Table S4), which was inserted to *nosP-cycB3* with the enzymes, *SbfI* and *AscI*. The 714 bp *GFP*-coding fragment was amplified with *pEGFP-N1* (Clontech) as a template (P6/P7 as primers, Table S4). Then, these two fragments of *GFP-* and *cycB3*-coding sequence were subcloned to *attB-cycB3P*, with *AscI* and *KpnI* as the connectors; finally the *attB-cycB3P-cycB3-gfp* (abbreviated as *cycB3P-cycB3-gfp*) construct was obtained.

### 4.2. Drosophila Stocks

*Oregon-R* was used as a wild-type strain. All fly stocks were cultured at room temperature on a standard *Drosophila* medium, except those with special requirements. The following strains were from Bloomington Stock Center: *cycB3*^2^ (#6635), *cycB3^EY^*^08012^ (#20013), *cycB3^L^*^6540^ (#10337), *cycB3* deficiency (abbreviated as *Df*) allele (#7679) and *nosP-gvp* (#31777). The *attP*-containing strain (#25709 and #25710) was used as the host for phiC31-mediated transformation [40]. The following lines were also used for experimentation: *DadP-GFP* and *bamP-GFP* [50]; *c587-gal4*, *neoFRT82B/TM3* and *hs-FLP*; *FRT82B*, *Ubi-GFP/TM3* [15].

### 4.3. Immunohistochemistry and Microscopy for Drosophila Ovary

Ovaries were prepared for immunohistochemistry, as described previously [7]. The following primary antibodies were used: rabbit anti-GFP (1:500, Abcam, Cambridge, MA, USA); mouse anti-Hts (1:100, DSHB, Iowa, IA, USA); mouse anti-Orb (1:200, DSHB); rabbit anti-Vasa (1:500, Yeasen, Shanghai, China). The following secondary antibodies were used at a 1:1000 dilution: goat anti-rabbit Alexa 488; goat anti-mouse Alexa 555 (Molecular Probes, Abcam, Cambridge, MA, USA). DAPI dye (Yeasen, Shanghai, China) was used to visualize cellular nuclei. All samples were examined with a Leica fluorescent microscope. All micrographs were taken with an Olympus confocal FV1000 microscope (Tokyo, Japan), and Z-stacks were obtained for presentation.

### 4.4. Quantitative Real-Time PCR (qPCR)

Total RNA was independently extracted from *Drosophila* ovaries with different genotypes (wild-type and *cycB3* mutants), using the Trizol Reagent (Sangon, Shanghai, China), and cDNA was synthesized, according to the manufacturer’s protocol (PrimeScript RT reagent Kit with gDNA Eraser, Takara, Dalian, China). Quantitative PCR was run on a CFX96 Touch (BioRad, Hercules, CA, USA) to measure total *cycB3* mRNAs, with *rp49* as a reference, according to the manufacturer’s protocol (SYBR Premix EX Taq™ II qPCR Kit, Takara, Dalian, China). The following primers were used in this assay (Table 3):

### 4.5. TUNEL Apoptotic Cell Detection

The apoptotic cell analyses were performed using the terminal deoxynucleotidyl transferase-mediated dUTP nick end-labeling (TUNEL) technique. The apoptotic GSCs from wild-type and *cycB3* mutant ovaries were analyzed according to the manufacturer’s protocol (TUNEL Assay Kit, Beyotime, Hangzhou, China) and the previous description [58].

### 4.6. Generating and Analyzing Marked Germline Clones

The FLP/*FRT*-mediated mitotic recombination technique was used to generate mutant GSCs and cyst clones, as described previously [15]. For example, to generate *cycB3* GSC clones, 2-day-old female flies, carrying the genotype *hs-flp/+*; *FRT82B*, *ubi-gfp/FRT82B*, *cycB3*^2^ (*hs-flp/+*; *FRT82B*, *ubi-gfp/FRT82B* as the wild-type control) were heat-shocked for 90 min at 37 °C, three times per day. After 5 consecutive days of heat-shock treatment, the flies were transferred to fresh food at room temperature, and ovaries were analyzed at days 2, 7, 14 after the last heat-shock treatment. GSC clones were identified by a lack of GFP expression, as well as from their attachment position to cap cells or terminal filament cells. Cyst clones were recognized by GFP-negative staining, as well as due to being far away from niche cells.

### 4.7. Statistical Analysis

A Chi-square test, or Student’s *t*-tests were used to assess relationships between allelic variables. The level of statistical significance was set at *p* < 0.05.

## 5. Conclusions

This study reveals that *Drosophila cyclin B3* (*cycB3*) plays a key role in the determination of the fate of GSCs fate. *cycB3* is required intrinsically for GSC maintenance. Our results indicate that *cycB3* is not involved in *Dad*-mediated regulating Bmp signaling, nor is it required for *bam* silencing, and it functions in a *bam*-dependent manner. The *cycB3* deficit fails to cause apoptosis in GSCs, and does not affect CB differentiation into oocytes. In addition, the overexpression of *cycB3* notably delays CB differentiation.

## Figures and Tables

**Table 1:** Phenotypic assay for *cycB3* mutation in *Drosophila* ovary.

Genotype	Age ^1^	Germaria				
		Empty	0 Germline Stem Cell (GSC) (Cysts Only)	1 GSC	2–3 GSCs	Total
*Oregon*	Day 1	0	0	1.4%	98.6%	146
	Day 7	0	0.6%	3.9%	95.5%	178
	Day 14	0.7%	1.4%	7.4%	90.5%	148
*cycB3*^2^*/cycB3*^2^	Day 1	2.9%	2.2%	5.8%	89.1%	274
	Day 7	26.6%	4.4%	29.6%	39.4%	203
	Day 14	42.1%	10.5%	24.8%	22.6%	420 *
*cycB3*^2^*/cycB3^EY^*^08012^	Day 1	1.9%	0.5%	3.3%	94.3%	209
	Day 7	22.5%	6.0%	25.8%	45.7%	267
	Day 14	38.8%	5.6%	28.0%	27.6%	340 *
*cycB3*^2^*/cycB3^L^*^6540^	Day 1	3.1%	3.6%	6.7%	86.6%	194
	Day 7	8.0%	1.9%	26.5%	63.6%	162
	Day 14	29.5%	15.8%	26.5%	28.2%	444 *
*cycB3^EY^*^08012^*/cycB3^L^*^6540^	Day 1	6.9%	0.9%	11.4%	80.8%	219
	Day 7	15.9%	6.0%	47.5%	30.6%	183
	Day 14	35.2%	13.5%	32.9%	18.4%	207 *
*cycB3*^2^*/Df*	Day 1	4.3%	3.8%	6.9%	85.0%	160
	Day 7	5.6%	11.7%	24.8%	57.9%	214
	Day 14	32.9%	20.8%	25.9%	20.4%	216 *

^1^ The days after eclosion. *Df*, Deficiency strain for *cycB3* gene. * *p* < 0.001 (*χ*^2^ test) when the total percentages of abnormal germaria (containing 1 GSC, 0 GSC and empty) from different *cycB3* mutant ovaries were compared with wild-types. Fourteen-day-old flies were selectively analyzed.

**Table 2:** Overexpression of *cycB3* dramatically increases the average number of Cystoblasts (CBs).

Genotype	The Average Number of GSCs (Mean ± SD)	*p* Value	The Average Number of CBs (Mean ± SD)	*p*-Value
*bamP-GFP* (*WT*)	2.1 ± 0.4 (*n* = 87)	-	1.1 ± 0.4 (*n* = 87)	-
*bamP-GFP*; *attB-cycB3-gDNA*	2.6 ± 0.9 (*n* = 83)	*p* < 0.05	2.8 ± 1.3 (*n* = 83)	*p* < 0.01
*bamP-GFP*; *nosP-cycB3*	3.0 ± 1.4 (*n* = 113)	*p* < 0.05	3.1 ± 1.7 (*n* = 113)	*p* < 0.01
*bamP-GFP*; *hsP-cycB3* (RT)	2.1 ± 0.5 (*n* = 89)	*p* > 0.05	1.1 ± 0.5 (*n* = 89)	*p* < 0.01
*bamP-GFP*; *hsP-cycB3* (HS)	2.2 ± 0.8 (*n* = 98)		3.2 ± 1.4 (*n* = 98)	

RT, room temperature; HS, heat-shock; SD, standard deviation; *n*, number of examined germaria; unpaired *t*-test, compared to *WT*.

**Table 3:** The primers in conducting qPCR experiment

Primer Names	Sequences of Primers
*cycB3*	5’-CAGTGCCCGAGGAAGTAGAG-3′ (sense)
	5’-GCATATAGTCGGCAATGGGG-3′ (antisense)
*rp49*	5’-CACTTCATCCGCCACCAGTC-3′ (sense)
	5’-CGCTTGTTCGATCCGTAACC-3′ (antisense)

## Supplementary Materials

Supplementary materials can be found at www.mdpi.com/1422-0067/19/1/298/s1.

## Abbreviations

*cycB3*	cyclin B3
GSC	germline stem cell
GFP	gfp fluorescent protein
CB	Cystoblast
*Df*	Deficiency strain of *cycB3* gene
qPCR	quantitative real-time PCR
*nosP-gvp*	nanosP-gal4:vp16
AHST	after heat-shock treatment
*UASp*	pattB-UAS
*hsP*	pattB-hsP
*nosP*	pattB-nosP
*Dad*	Daughters agaist Dpp
TUNEL	Terminal deoxynucleotidyl transferase(TdT)-mediated dUTP Nick End Labeling
*WT*	wild-type
