# Supplementary material for: Cyclin B3 Deficiency Impairs Germline Stem Cell Maintenance and Its Overexpression Delays Cystoblast Differentiation in Drosophila Ovary
Source: Int J Mol Sci. 2018 Jan 19;19(1):298. doi: 10.3390/ijms19010298 (PMC5796243; doi:10.3390/ijms19010298)
Supplement: Supplementary file 1 [file ijms-19-00298-s001.pdf]

Table S1. The rescue assay of *cycB3* mutants by the transgenic line of P{*attB-cycB3-gDNA*}

Genotype	Age[1]	Germaria				
		**Empty**	**0 GSC (cysts only)**	**1 GSC**	**2-3 GSCs**	**Total**
cycB3²/cycB3²	day1	3.0%	2.0%	5.6%	89.4%	198
	day14	41.8%	11.6%	23.3%	23.3%	146
attB-cycB3-gDNA;	day1	2.1%	0	1.6%	96.3%	190
cycB3²/cycB3²	day14	0	0.9%	0.9%	98.2%	108*
cycB3²/cycB3EY08012	day1	2.5%	1.7%	2.5%	93.3%	120
	day14	24.0%	5.3%	24.0%	46.7%	167
attB-cycB3-gDNA;	day1	0	2.5%	1.0%	96.4%	196
cycB3²/cycB3EY08012	day14	0	0	2.6%	97.4%	193*

[1]the days after eclosion. *P<0.001* (χ^2 test) when the total percentages of abnormal germaria (containing 1 GSC, 0 GSC and empty) from different *cycB3*-rescuing ovaries were compared with their corresponding mutants. 14-day-old flies were selectively analyzed.

Table S2. *cycB3* is intrinsically required for GSC maintenance in *Drosophila* ovary

Genotype	Age[1]	Germaria				
		**Empty**	**0 GSC (cysts only)**	**1 GSC**	**2-3 GSCs**	**Total**
cycB3²/cycB3²	day1	2.4%	2.4%	7.0%	88.2%	212
	day14	40.3%	12.9%	23.7%	23.1%	186
UASp-cycB3;nosP-gvp,	day1	1.0%	0	3.3%	95.7%	210
cycB3²/cycB3²	day14	0	0.7%	3.0%	96.3%	134*
nosP-cycB3;	day1	0	0	2.2%	97.8%	312
cycB3²/cycB3²	day14	1.2%	0.5%	1.2%	97.1%	347*
cycB3²/cycB3EY08012	day1	2.0%	0.5%	5.0%	92.5%	200
	day14	34.7%	13.5%	26.5%	25.2%	230
UASp-cycB3/nosP-gvp;	day1	0	0.4%	2.1%	97.5%	237
cycB3²/cycB3EY08012	day14	0	0	4.0%	96.0%	125*
nosP-cycB3;	day1	0	0	2.7%	97.3%	339
cycB3²/cycB3EY08012	day14	0	0	1.9%	98.1%	159*
c587-gal4;UASp-cycB3;	day1	4.5%	0.8%	9.1%	85.6%	132
cycB3²/cycB3²	day14	32.9%	5.1%	32.9%	29.1%	237#
c587-gal4;UASp-cycB3;	day1	2.3%	0	8.7%	89.0%	219
cycB3²/cycB3EY08012	day14	44.8%	10.5%	17.5%	27.2%	257#

[1]the days after eclosion. *P<0.001* (χ^2 test) when the total percentages of abnormal germaria (containing 1 GSC, 0

GSC and empty) from different *cycB3*-rescuing ovaries were compared with their corresponding mutants. $^{\#}P>0.05$ (χ^2 test) when the total percentages of abnormal germaria from cycB3-rescuing ovaries were compared with their corresponding mutants. 14-day-old flies were selectively analyzed.

Table S3. The analysis of the cell death in *cycB3* mutant ovaries.

**Genotype**	**The percentages of apoptotic GSCs between wild type and *cycB3* mutant fly ovaries**
Oregon-R	1.4% (N=210)
cycB3^2/*cycB3*^{2}	1.8% (N=223)
FRT control	1.5% (N=201)
FRT cycB3^2	1.0% (N=197)

N, the total number of GSCs examined.

Table S4. Primers used for generating *cycB3* transgenic vectors.

**No.**	**Primer Sequences**	**Restriction enzymes (underlined)**
P1	5′-AAACCTGCAGGCCAATGTTGCCCTAAGAAATTCATAATGGG-3′(forward)	*SbfI*
P2	5′-TTTGGTACCTTAACCTTTGACATCTAGGAGTTTATGCTC-3′(reverse)	*KpnI*
P3	5′-TTTGGCGCGCCATGGCGCCCACAAAAGCAACAAC -3′ (forward)	*AscI*
P4	5′-TTTGGTACCCTACGACAGATTGCTTTCGTTC-3′ (reverse)	*KpnI*
P5	5′-TTTGGCGCGCCCGACAGATTGCTTTCGTTCAGGTCAAG-3′(reverse)	*AscI*
P6	5′- AAAGGTACCGCCGCTGTGGGCAAAGGA-3′(forward)	*KpnI*
P7	5′- TTTGGTACCTTACTTGTACAGCTCGTCCATGCCG-3′(reverse)	*KpnI*
